# Experiences of US Clinicians Contending With Health Care Resource Scarcity During the COVID-19 Pandemic, December 2020 to December 2021

**DOI:** 10.1001/jamanetworkopen.2023.18810

**Published:** 2023-06-16

**Authors:** Catherine R. Butler, Aaron G. Wightman, Janelle S. Taylor, John L. Hick, Ann M. O’Hare

**Affiliations:** 1 Division of Nephrology, Department of Medicine and the Kidney Research Institute, University of Washington, Seattle; 2 Nephrology Section, Hospital and Specialty Medicine and Seattle-Denver Health Services Research and Development Center of Innovation, Veterans Affairs Puget Sound Health Care System, Seattle, Washington; 3 Treuman Katz Center for Pediatric Bioethics, Seattle Children’s Hospital, Seattle, Washington; 4 Department of Pediatrics, University of Washington School of Medicine, Seattle; 5 Department of Anthropology, University of Toronto, Toronto, Ontario, Canada; 6 Hennepin Healthcare, University of Minnesota, Minneapolis

## Abstract

**Importance:**

The second year of the COVID-19 pandemic saw periods of dire health care resource limitations in the US, sometimes prompting official declarations of crisis, but little is known about how these conditions were experienced by frontline clinicians.

**Objective:**

To describe the experiences of US clinicians practicing under conditions of extreme resource limitation during the second year of the pandemic.

**Design, Setting, and Participants:**

This qualitative inductive thematic analysis was based on interviews with physicians and nurses providing direct patient care at US health care institutions during the COVID-19 pandemic. Interviews were conducted between December 28, 2020, and December 9, 2021.

**Exposure:**

Crisis conditions as reflected by official state declarations and/or media reports.

**Main Outcomes and Measures:**

Clinicians’ experiences as obtained through interviews.

**Results:**

Interviews with 23 clinicians (21 physicians and 2 nurses) who were practicing in California, Idaho, Minnesota, or Texas were included. Of the 23 total participants, 21 responded to a background survey to assess participant demographics; among these individuals, the mean (SD) age was 49 (7.3) years, 12 (57.1%) were men, and 18 (85.7%) self-identified as White. Three themes emerged in qualitative analysis. The first theme describes isolation. Clinicians had a limited view on what was happening outside their immediate practice setting and perceived a disconnect between official messaging about crisis conditions and their own experience. In the absence of overarching system-level support, responsibility for making challenging decisions about how to adapt practices and allocate resources often fell to frontline clinicians. The second theme describes in-the-moment decision-making. Formal crisis declarations did little to guide how resources were allocated in clinical practice. Clinicians adapted practice by drawing on their clinical judgment but described feeling ill equipped to handle some of the operationally and ethically complex situations that fell to them. The third theme describes waning motivation. As the pandemic persisted, the strong sense of mission, duty, and purpose that had fueled extraordinary efforts earlier in the pandemic was eroded by unsatisfying clinical roles, misalignment between clinicians’ own values and institutional goals, more distant relationships with patients, and moral distress.

**Conclusions and Relevance:**

The findings of this qualitative study suggest that institutional plans to protect frontline clinicians from the responsibility for allocating scarce resources may be unworkable, especially in a state of chronic crisis. Efforts are needed to directly integrate frontline clinicians into institutional emergency responses and support them in ways that reflect the complex and dynamic realities of health care resource limitation.

## Key Points

**Question:**

How were health care crisis conditions during the second year of the COVID-19 pandemic experienced by frontline clinicians?

**Findings:**

In this qualitative study of 23 clinicians, participants reported that official crisis declarations had little impact on their lived clinical experience. In the absence of institutional support, many clinicians were left to allocate scarce resources and adapt practices in the moment, which could have contributed to a waning sense of motivation and purpose as the pandemic persisted.

**Meaning:**

These findings suggest that institutional plans to protect frontline clinicians from the responsibility of allocating scarce resources could be unworkable, arguing for greater integration of these clinicians into disaster response, especially in protracted crises.

## Teaser

This qualitative study assesses clinician experiences during periods of extreme scarcity of health care resources in the second year of the COVID-19 pandemic.

## Introduction

From the earliest days of the COVID-19 pandemic, there was a strong focus on developing strategies to respond to extreme crisis conditions in which the demand for scarce life-saving resources, such as intensive care unit (ICU) beds and mechanical ventilators, outstripped the supply. Many states and institutions worked to develop algorithms for rationing scarce resources in what was envisioned to be an exceptional, clearly demarcated, and temporary state of crisis.^1^ These planning processes drew on well-established emergency response frameworks^2,3,4^ and envisioned a coordinated regional response and standardized process of scarce resource allocation across a population of patients.^5,6^ Most drew a sharp distinction between system-wide rationing procedures designed to promote effective and equitable allocation of scarce resources and the work of frontline clinicians that typically focuses on providing the best care for individual patients. Shifting responsibility for resource allocation to system-level processes was also seen as a way to relieve clinicians of personal and legal responsibility related to providing care under conditions of resource scarcity.^7,8,9^

While early waves of the COVID-19 pandemic in the US prompted many states and institutions to engage in emergency planning,^10,11,12,13^ it was generally not until the second year of the pandemic that ongoing episodes of widespread resource limitation prompted some US states to enact regional policy responses.^14,15^ While these formal declarations of crisis garnered substantial media attention,^16,17,18,19^ it is unclear how crisis conditions were experienced by frontline clinicians. Building on work describing clinician experiences during the first year of the pandemic,^20,21^ we sought to elicit clinician experiences during these periods of extreme resource scarcity later in the pandemic.

## Methods

We conducted a qualitative, interview-based study to understand the experiences of US clinicians caring for patients in crisis conditions during the second year of the COVID-19 pandemic. The University of Washington Institutional Review Board approved this study and authorized verbal consent in lieu of written informed consent. Consolidated Criteria for Reporting Qualitative Research (COREQ) were used to guide the methodologic approach.

### Data Collection and Participant Recruitment

Clinicians working in states with official declarations and/or where media coverage suggested crisis conditions^14,16,18,19^ were initially identified by searching institutional websites and through direct referrals from colleagues. We then used a snowball recruitment technique in which participants were invited to provide contact information for colleagues who might be interested in participating in the study.^22^ We purposefully directed recruitment to enhance an understanding of emerging concepts as the data were analyzed.^23^

A semistructured interview guide (Box) was developed by 2 investigators who are physician scientists, health services researchers, and practicing nephrologists in Seattle, Washington (C.R.B. and A.M.O.), one of whom is also a bioethicist (C.R.B.). The guide included open-ended questions to elicit clinicians’ experiences pertaining to clinical care, professional interactions, and institutional policies during the second year of the pandemic. Clinicians completed a one-on-one 30- to 60-minute video or telephone interview with a team member who has training and experience in qualitative interviewing (C.R.B.). The interview guide was iteratively refined during the course of the study to allow for elaboration of emerging themes. Interviews were audio-recorded with permission and transcribed verbatim. Participants also completed an online background survey (REDCap^24^) with questions about their demographic characteristics and practice experience. Race and ethnicity data were collected as these characteristics may shape personal experiences. Participants self-reported race as American Indian or Alaska Native, Asian, Black or African American, Native Hawaiian or Other Pacific Islander, White, or more than 1 race and ethnicity as Hispanic or Latino or non-Hispanic or non-Latino. All interviews were conducted between December 28, 2020, and December 9, 2021.

Box. **Example Interview Questions**

**Questions**

- What has it been like for you to work during the COVID-19 pandemic?
- Have you had to change how you take care of patients? In what ways?
- Do you have examples of situations in which you had difficulty getting patients the care that they needed?
- Have you encountered instances of staffing limitation?
- Have you been involved in developing plans for responding to limited resources or staffing shortages?
- Is there anything that we haven’t covered that you’d like to bring up?

**Probes**

- Can you tell me more about [word participant used]?
- Can you give me an example?
- What was that like?
- What has been similar or different than in the past?
- What went well? What was difficult?

### Qualitative Analysis

Two investigators (C.R.B. and A.M.O.) independently reviewed and openly coded phrases (ie, labeled conceptual constructs) in interview transcripts until reaching theoretical saturation (ie, the point at which few new constructs were identified with additional coding).^23,25,26^ One team member (C.R.B.) coded all remaining transcripts to identify additional exemplar quotations. We used the constant comparative method, in which analysts compare constructs as these emerge from successive interview transcripts and consider how these constructs might expand on, contradict, and/or support emerging themes and conceptual schema.^23,27^ Two investigators (C.R.B. and A.M.O.) worked together to collapse codes into groups with related meanings, identify relationships between code groups, and develop broader thematic categories, returning to the transcripts as needed to confirm that emerging themes and subthemes were grounded in the interview transcripts.^26,28^ All investigators (a pediatric nephrologist and bioethicist [A.G.W.], a medical anthropologist [J.S.T.], and an emergency medicine physician practicing in Minneapolis, Minnesota, who participated in state and national crisis care planning [J.L.H.]) reviewed exemplar quotations and contributed to iterative refinement of thematic descriptions and categories. Atlas.ti, version 8 (Scientific Software Development GmbH), was used to organize and store text and codes.

## Results

Twenty-three clinicians participated in this study, representing a range of clinical roles, perspectives, and experiences. The mean (SD) age of the 21 study participants who responded to the background survey was 49.0 (7.3) years, 9 (42.9%) were women, and 12 (57.1%) were men. Three participants (14.3%) were Asian or South Asian and 18 (85.7%) were White; 5 participants (23.8%) were Hispanic or Latino and 16 (76.2%) were non-Hispanic or non-Latino. Most of the 23 participants were physicians (21 [91.3%]), were working in intensive care settings (14 [60.9%]), and were practicing at academic (10 [43.5%]) or community (9 [39.1%]) medical centers in 4 US states (California, Idaho, Minnesota, and Texas) (Table 1).

**Table 1.** Participant Characteristics^a^

Characteristic	No. (%)
Age, mean (SD), y (n = 21)	49.0 (7.3)
Sex (n = 21)	
Women	9 (42.9)
Men	12 (57.1)
Race (n = 21)	
Asian or South Asian	3 (14.3)
White	18 (85.7)
Ethnicity (n = 21)	
Hispanic or Latino	5 (23.8)
Non-Hispanic or non-Latino	16 (76.2)
Type of institution (n = 23)	
Academic	10 (43.5)
Community	9 (39.1)
Private	3 (13.0)
Other	1 (4.3)
Clinical site (n = 23)^b^	
Clinic or outpatient	8 (34.8)
Acute care	11 (47.8)
Intensive care	14 (60.9)
Emergency department	2 (8.7)
Research	4 (17.4)
Institutional setting (n = 23)	
Urban	20 (87.0)
Rural	3 (13.0)
Clinical role (n = 23)	
Registered nurse	2 (8.7)
Physician	21 (91.3)
Time worked in health care setting, mean (SD), y (n = 21)	20.8 (7.1)
State (n = 23)	
California	11 (47.8)
Texas	3 (13.0)
Minnesota	5 (21.7)
Idaho	4 (17.4)
Has there been a declaration of crisis capacity in your state? (n = 21)	
Yes	15 (71.4)
No	4 (19.0)
Do not know	2 (9.5)

^a^ Twenty-one of 23 participants responded to the background survey. For those participants who did not complete the survey, background information that appeared in the interview transcript was collected.

^b^ Respondents could choose more than 1 answer.

Three themes, each with several subthemes, pertaining to clinicians’ experiences of providing care under conditions of extreme resource limitation during the second year of the COVID-19 pandemic emerged from qualitative analysis: (1) isolation; (2) in-the-moment decision-making; and (3) waning motivation. Exemplar quotations for each of these themes are included in Table 2.

**Table 2.** Themes and Example Participant Quotations

Participant’s work context	Quotation
**Theme 1: isolation**	
Differing definitions of crisis	
Minnesota community institution	Almost nobody has been in [an official] crisis, but we’ve been in crisis. And you don’t declare formal crisis in one sort of system of measurement, and yet you’re not well. You’re sending a very, to me, a bad message, and inconsistent message.
California academic institution	They were creating a bunch of beds that have ICU capacity. But they weren’t really getting enough input from the frontline providers that would be able to tell you things like…this particular room will be a problem…because there’s all of these windows such that we can’t see any of the monitors…but this room over here actually is perfect.
Idaho community institution	All the intensivists felt [a crisis declaration] probably ought to happen statewide based on what we were experiencing on the ground. But…the administrators from our institutions did not feel we were in crisis standards.…The disconnect between what is said from administrative people and what is the experience on the ground.…At best, it feels like I’m being dismissed for my experience…at worst, I’m being gaslit with how they’re rewriting it.
Limited view	
California private institution	You get these pages.…“There’s 9 ICU patients waiting for an ICU bed.”…You wonder who else is going to be waiting if you move [your patient] to the ICU room.…I don’t know that situation, I can only advocate for the patients I’m taking care of.
California academic institution	We said, once we’re off service, we’ll come back just to set up the cyclers, because that support is needed.…But then other [physicians] were like, “Why can’t the nurses do it?”…It was just that complete lack of frame of reference.
California academic institution	Normally…I would say, “Hey…I think this patient needs CRRT.”…Now, I just go to the nephrology staff and, “Hey, I think this patient needs volume off. How can you get me volume off?”…Because I don’t understand the resource limitations that they’re seeing from their nursing and machines.…I don’t always understand their limitations.
California academic institution	[Fatigued clinicians are asking] “Are the other services in the hospital, how are they going to help me out?”…Not all of the problems in the hospital are visible to you, we tend to be a little bit myopic.
Responsibility without power	
Idaho community institution	The triage piece that was supposed to be in place never materialized.…We wrote the [crisis standards of care] guidelines in such a way that that distress was supposed to be distributed…[but] it fell pretty squarely on the point-of-care providers.…It was always the frontline person who was triaging, and quite frankly rationing…I had to do all of this by myself [and] I feel psychically distressed by it.
California academic institution	I got the sense that nobody was in charge.…I had to remind myself numerous times a day, ok, nobody is coming to our rescue. If we don’t do this right now, it’s just not going to happen.…It was the anxiety over the sort of vacuum of leadership.
Idaho community institution	[Patients] get stuck out in the rural hospitals.…[I say to the transfer center] “Find a way to get this patient here. Put him in the hall. I don’t care, we can find a way to dialyze him.”…And the transfer center people [say] “Let me talk to my supervisor.” Two hours later: “Supervisor says no.” So, you know, I’m pretty sure patients are dying out there.
**Theme 2: in-the-moment decisions**	
Official crisis declaration could be dissociated from clinical experience	
Idaho community institution	Everybody who wanted to be full code got at least a run of CPR. Regardless of their prognosis, even though we’re in crisis standards, even though epinephrine was on shortage.…We didn’t get any guidance from our office, so we were having to make these decisions on our own, which we weren’t supposed to.
Idaho community institution	I don’t believe that the crisis standards carry…a lot of weight from a liability standpoint. You know, I think that would be worked out in a court and…I’d rather not have that experience.
Idaho community institution	We never activated our triage committee to look at all the patients that might need resources and where they should stack in any sort of framework. It ended up coming, becoming either first-come, first-served or who was just lucky enough to get that first phone call in when a bed came open.
Clinical judgment applied to resource allocation	
California academic institution	Usually you might be more willing to try CRRT for 1 or 2 days before we say no. But here I was much more willing to be like, we won’t even try.…If this is all futile and also delays somebody else’s treatment by 4 hours, I’m like, this just doesn’t make sense.
Minnesota other type of institution	I had 2 patients who were on trachs, both waiting for LTAC, an LTAC bed opened up, I picked the one who was like 50, and I sent the 80-year-old to the floor. And that was me, in that moment, deciding I pick you.…There wasn’t a ton of thought. I mean, the younger patient clearly had better rehab potential than the older one.
Idaho community institution	We have put ICU patients in the PACU in addition to non-ICU areas that have been retrofitted with monitors, [but] we have not cared for any ICU patients without adequate monitoring.…We did not want to walk in on anyone who was dead. There are certain minimal standards.
Minnesota academic institution	We talked about what our contingency plans might be, like 12 hours on, 12 hours off [of continuous dialysis].…Well, it is rationing. Well, no, that really wouldn’t be rationing if we needed to spread it out to try to provide care to more people than we had machines.
California academic institution	I can’t really point to any example where I could have an identifiable [patient] outcome difference…[but] you kind of wonder, if a patient was, instead of being in a converted stepdown unit, or in a converted ED bay, and they were back up in my ICU…would their care be a little bit different?
Moral ambiguity	
Idaho community institution	That triage piece…trying to save the most lives…we certainly didn’t have a scoring system that would take into account all of these variables…[for example] a less-ill patient who was 4 hours away in a critical access hospital was in bigger trouble than a more sick person who was already with me.…That was tough to figure out the calculus, the formula for what was right.
Minnesota community institution	I called it ‘fuscarcity’…to send you to ICU with your end-stage cancer with comorbidities, it feels like it may be futile, it always kind of does. But now you have…this added pressure of scarcity to our existing ethical and moral dilemmas.
**Theme 3: waning motivation**	
Selflessness wearing thin	
California private institution	They feel like they’re getting taken advantage of.…You throw them in a pandemic where everybody feels morally obligated to work more, and to do more with less, you see people with the best intentions who want to do good work get really depressed and hopeless.
Minnesota community institution	It’s maybe the valorization or the calling. We’re here to protect our community, we’re here to serve, and to keep people from harm, to keep people from dying. That culture, it’s a good culture, [but] it’s just circumstances we never faced before.
Idaho community institution	I don’t think that in any foreseeable future would I take even 2 weeks [off] because of the burden that I would leave for the rest of my group.
Idaho community institution	Most of us go [to medical school] because of a sense of trying to do good in the world. So, to add on this distrust and disappointment, antagonism [among patients]. When you’re having to worry about if somebody’s going to come in and beat you up and try to shoot you because you’re not doing what they think is appropriate therapy.
Narrowing clinical roles	
California academic institution	To maximize physician resources…[it works to take an] assembly-line approach to patient care, where you maximize what people are really good at.…[But], in becoming that efficient, for me anyway, it removes a lot of the joy.…If you’re not able to keep what you’re doing somewhat light and sort of remind yourselves that this is anchored to being a human being, this becomes an intolerable job after a while.
California academic institution	[The trainees] had a very, very high level of burnout.…They were the ones who were writing the notes.…You remove the whole doctoring part where you basically are turned into this glorified administrative assistant.…The sense of feeling helpless was really difficult.
California academic institution	Never in my career had I ever thought about physicians in an inpatient setting as a resource.…If you strip away everything, what does this person add to patient care?…It was a little bit dehumanizing. Because it ignored a lot of the emotional side.
Staff as a limited resource	
Texas community institution	We ran out of oxygen. So they had to come and overnight figure out a way to bring in more tanks…doctors, administration, RNs, everybody we knew, plus people coming in like IT people, our mechanics were coming in working day and night trying to fix our hospital to where we could be able to help these people.
Idaho community institution	There’s a limit to how many patients I can see in a day.…It wasn’t physically possible to do, and then there’s rationing of *me* going on.
California academic institution	Bad call nights or long ICU stretches…it’s what we’ve all done.…The hard part is you know that tomorrow, the day after, that break is not really going to be there. And I think that’s where it starts to drag on you emotionally.…You can power through anything for a short period of time.…[but] this is not a sprint, this is a marathon, and I hate long-distance running.

Abbreviations: CPR, cardiopulmonary resuscitation; CRRT, continuous renal replacement therapy; ED, emergency department; ICU, intensive care unit; IT, information technology; LTAC, long-term acute care facility; PACU, postanesthesia care unit; RN, registered nurse.

### Theme 1: Isolation

#### Differing Definitions of Crisis

Clinicians described the complexity of how resource and staffing limitations manifested in real time and the difficulty of determining what would or should constitute a crisis. As one clinician put it, “almost nobody has been in [an official] crisis, but we’ve been in crisis. And you don’t declare formal crisis…yet you’re not well.” Standard measures of resources, such as the number of ICU beds, were not always meaningful when clinicians’ ability to care for patients was also shaped by dynamic clinical needs and varied availability of particular material and staffing resources. Several clinicians believed that they had been working under crisis conditions long before (or in the absence of) formal declarations and cited a “disconnect between what is said from administrative people and what is experienced on the ground,” which could leave clinicians feeling unsupported, “dismissed,” or even “gaslit” by leadership.

#### Limited View

Clinicians described what could be a myopic focus on their immediate environment as they were confronted by clinical work that was fast paced, rapidly changing, and all-consuming. They had a limited view on the challenges faced by other clinical teams, institutional processes, and global resource constraints, which hindered their ability to coordinate and distribute resources across groups of patients. One clinician reflected, “You wonder who else is going to be waiting if you move this person to the ICU room…[but] I don’t know that situation, I can only advocate for the patients I’m taking care of.” Clinicians also cited a “complete lack of frame of reference” among colleagues who did not have first-hand experience caring for patients, which made it difficult to coordinate care and strained professional relationships. An intensivist described feeling limited in how they could participate in decision-making about dialysis modality with nephrology consultants because “I don’t understand the resource limitations that they’re seeing.” Others wondered why they were not receiving more help from other clinical teams. “[You’re thinking] ‘How are they [other clinicians] going to help me out?’…[But] not all of the problems in the hospital are visible to you.”

#### Responsibility Without Power

In the absence of systematic institutional strategies to guide practices, ultimate responsibility for resource allocation and practice adaptation fell to the frontline clinicians, despite their limited power over the system-level factors that were shaping this care. “The triage piece…never materialized…distress was supposed to be distributed…[but] it was always the frontline person who was triaging, and quite frankly rationing.” Recognizing that “nobody is coming to our rescue,” clinicians described going to considerable lengths to try to mobilize needed institutional resources. At the same time, most lacked both the bandwidth and authority needed to coordinate efforts across teams or institutions. One clinician described struggling to coordinate transfer from a critical access hospital for a patient who needed life-saving dialysis treatment and how their efforts were met with insurmountable administrative barriers: “the transfer center people [say] ‘Let me talk to my supervisor.’ Two hours later: ‘Supervisor says no.’ So…people are dying out there.”

### Theme 2: In-the-Moment Decision-making

#### Official Crisis Declaration Could Be Dissociated From Clinical Experience

Some clinicians who had practiced under official declarations of crisis saw these as largely outward-facing statements intended to secure external resources or capture public attention. Notably absent were any explicit changes in the way that health care resources were being allocated in the clinical setting or in guidance from institutional leadership. One clinician described a challenging situation in which “epinephrine was on shortage….We didn’t get any guidance from our office, so we were having to make these decisions [regarding performing cardiopulmonary resuscitation] on our own, which we weren’t supposed to.” Clinicians saw no evidence of the explicit rationing procedures or liability protections that had been specified in institutional planning processes “We never activated our triage committee….[Patients were treated] first-come, first-served, or who was just lucky enough to get that first phone call when a bed came open.”

#### Clinical Judgment Applied to Resource Allocation

Under conditions of resource scarcity, clinicians were generally less willing to offer therapies that were believed to be of marginal benefit. As one clinician put it, “Usually you might be more willing to try [kidney dialysis] for 1 or 2 days…[but] if this is all futile and also delays somebody else’s treatment…this just doesn’t make sense.” Decision-making about how to allocate resources was typically guided in real time by individual clinical judgment and experience. One clinician described the process of assigning rehabilitation beds as “in that moment.…There wasn’t a ton of thought…the younger patient clearly had better rehab potential.”

What constituted acceptable changes to practice shifted with the degree of resource limitation, but clinicians described maintaining some universal minimal standard of care and tended not to view what they were doing to conserve and prioritize resources as overt rationing. Nonetheless, they expressed qualms about the lack of standardization and lower quality of the care that they were providing. Many were conscious of their limited view on the downstream or indirect effects of their own medical decisions and of broader practice changes on patient outcomes.

#### Moral Ambiguity

Many clinicians provided examples of morally distressing situations in which clinical reasoning and available guidelines proved woefully inadequate to inform decisions. For example, one intensivist described grappling with decisions about whether to prioritize care for the large number of patients seeking admission through the emergency department vs accepting transfers from smaller hospitals: “We certainly didn’t have a scoring system that would take into account all of these variables…a less-ill patient who was 4 hours away in a critical access hospital was in bigger trouble than a more sick person who was already with me.…That was tough to figure out the calculus, the formula for what was right.” The pressure of resource scarcity also added layers of complexity to more familiar ethical dilemmas, such as whether to provide treatments that clinicians considered marginally beneficial or futile.

### Theme 3: Waning Motivation

#### Selflessness Wears Thin

Many participants spoke of the selflessness and dedication of their colleagues early in the pandemic but also commented about how a moral imperative to sacrifice could contribute to professional burnout as the pandemic persisted. “The valorization or the calling…we’re here to serve…it’s a good culture, [but] it’s just circumstances we never faced before.” Some participants conveyed that setting one’s own limits or taking breaks could feel like an abandonment of patients or coworkers, but volunteerism and trust in colleagues seemed to fade over time. Additionally, misalignment between clinicians’ own goals and values with those of institutional leadership as well as strained relationships with patients and families reduced their satisfaction with work. One participant commented: “Most of us go [to medical school] because of a sense of trying to do good in the world,” so it was demoralizing “to add on this distrust and disappointment, antagonism [among patients].”

#### Narrow Clinical Roles

Many of the clinicians with whom we spoke described a shift toward an “assembly-line” approach to care whereby clinicians were assigned to narrowly defined tasks such as medical record documentation or overseeing specific procedures. While this approach might improve the efficiency of care in the short term, over time a narrow focus on task completion limited opportunities for professional growth and job satisfaction and could feel dehumanizing. “Becoming that efficient, for me anyway, it removes a lot of the joy…[it] becomes an intolerable job after a while.” While they tended to agree with the importance of prioritizing treatments focused on life extension when resources were scarce, clinicians also saw how such practices could involve tradeoffs in how well they could support patient dignity and the needs of family members, especially at end of life.

#### Staff as a Limited Resource

As the pandemic went on, staffing capacity was stretched thin. Many of the clinicians we interviewed found the notion of denying treatments due to staffing shortages to be unthinkable, preferring to extend themselves beyond any prior expectations to fill gaps in care. However, there were limits, and as one clinician explained, “It wasn’t physically possible to do, and then there’s rationing of *me* going on.” While their work could be physically demanding, what was often most difficult was the ongoing psychological strain with no end to the pandemic in sight: “You can power through anything for a short period of time…[but] this is not a sprint, this is a marathon.”

## Discussion

For clinicians practicing under conditions of extreme resource scarcity during the second year of the COVID-19 pandemic, official declarations of crisis often bore little relation to their own clinical experience and did not prompt the kinds of systematic shifts in resource allocation that had been anticipated. In the absence of institutional support, practice guidance, or knowledge of the broader institutional context, clinicians used their clinical judgment to prioritize treatments among the individual patients under their care. As the pandemic persisted, the unfulfilling and morally distressing nature of their work eroded the sense of mission and purpose that had motivated their efforts earlier in the pandemic.

When planning for crisis, separation of system-level resource allocation processes from bedside clinical care was seen as a critical step in guarding against moral distress among clinicians and promoting fairness and equity. However, the accounts of clinicians practicing under conditions of extreme resource limitation during the second year of the pandemic suggest that such a sharp delineation may be neither possible nor desirable.^29,30^ The experiences of the clinicians with whom we spoke resonate with Sherri Fink’s harrowing account of the breakdown in system-level processes at Memorial Hospital in New Orleans in the wake of Hurricane Katrina.^31^ As Fink described, “In the end, with systems crashing and failing, what mattered most and had the greatest immediate effects were the actions and decisions made in the midst of a crisis by individuals.”^31^ In the absence of clear action by institutional and regional leadership, the clinicians with whom we spoke were left with the responsibility for responding to crisis conditions without support or protection. They leveraged their clinical understanding of the relative urgency and value of different treatments for individual patients to make allocation decisions but lacked tools and information to know whether these decisions were effective and equitable on a population level.^32,33^ Based on the complex and dynamic challenges experienced during the COVID-pandemic, we suspect that even the best system planning is unlikely to obviate the need for some individualized bedside decision-making by clinicians about how to utilize limited resources, highlighting the critical importance of liability protection^7^ and clinician training in ethical principles^34^ and bias reduction.^35,36^ However, our findings also speak to the importance of providing frontline clinicians with a seat at the table in institutional operations and planning processes^37^ to inform rapid system response and iterative adaptation to evolving conditions.^15,38^

As traditionally defined, the term *crisis* refers to an extraordinary and temporary—rather than persistent and ongoing—state of affairs. In his work on Médecins sans Frontières, an organization designed to provide temporizing interventions during emergent humanitarian crises, Redfield^39^ describes the particular challenges of prolonged states of crisis where the organization might be able to “preserve existence while deferring the very dignity and redemption it seeks.” Our interviews with clinicians practicing under conditions of resource scarcity during the second year of the pandemic bear some parallels to the state of chronic crisis that Redfield describes. Early in the pandemic, there was general agreement on a singular goal of maximizing the number of lives saved^40,41,42^ (though even this early framing quickly met with concerns about equity^43,44,45,46,47^). However, the experiences of the clinicians interviewed for our study suggest that the ongoing nature of the pandemic emergency called for a more expansive and complex set of goals to also include providing best care for individual patients, allocating resources equitably across communities and populations, sustaining the clinical workforce,^48,49^ and maintaining institutional viability. Ultimately, existing models of responding to health care resource limitation proved too narrow to adequately address the multiple, far-reaching, and unanticipated impacts of a prolonged pandemic.

### Limitations

This study has some limitations. In interpreting our results, it is important to recognize that this study represents the experiences of only a select group of US clinicians working during the second year of the COVID-19 pandemic. Our findings might not be transferable to clinicians working in US regions not represented in this study or to nonphysician health care professionals. While the study was designed to capture diverse perspectives, a snowball recruitment strategy may have limited recruitment to people within social networks who may share similar values. Most participants identified as White and non-Hispanic; thus, our findings might not represent the experiences of Black clinicians as well as those of other minority racial and ethnic backgrounds.

## Conclusions

The findings of this qualitative study among clinicians working under conditions of extreme health care resource scarcity during the second year of the COVID-19 pandemic reflect the challenges of operating under dynamic, evolving, and persistent crisis conditions. These experiences illustrate how many theoretical plans intended to protect frontline clinicians from decisions about resource allocation were ultimately unworkable, leaving clinicians in the difficult position of having to allocate scarce resources and adapt care as best they could. Collectively, our findings highlight the importance of integrating frontline clinicians into institutional planning and operations when dealing with the realities of chronic resource limitation.

## Notes

Supplement.
Data Sharing Statement
