# Supplementary material for: Experiences of US Clinicians Contending With Health Care Resource Scarcity During the COVID-19 Pandemic, December 2020 to December 2021
Source: JAMA Netw Open. 2023 Jun 16;6(6):e2318810. doi: 10.1001/jamanetworkopen.2023.18810 (PMC10276299; doi:10.1001/jamanetworkopen.2023.18810)
Supplement: Supplement. — Data Sharing Statement [file jamanetwopen-e2318810-s001.pdf]

Data Sharing Statement

Butler. Experiences of US Clinicians Contending With Health Care Resource Scarcity During the COVID-19 Pandemic, December 2020 to December 2021. *JAMA Netw Open*. Published June 16, 2023. doi:10.1001/jamanetworkopen.2023.18810

Data
Data available: No
